# Settling in New Zealand’s Small Towns: Experiences of Minority Ethnic Immigrants

**DOI:** 10.1007/s12134-023-01044-6

**Published:** 2023-05-05

**Authors:** Ashraful Alam, Etienne Nel, Douglas Hill, Hannah Bulloch

**Affiliations:** 1 School of Geography, University of Otago, Dunedin, New Zealand; 2 Faculty of Architecture, Building and Planning, The University of Melbourne, Melbourne, Australia; 3 School of Social Sciences, University of Otago, Dunedin, New Zealand

**Keywords:** New Zealand, Small town, Migration, Ethnic minority, Settlement experience

## Abstract

Small town New Zealand has, in recent years, experienced an increasing number^1^ and diversity of immigrants, with visible yet under-researched impacts for less populated regions that historically have been dominated by Pākehā (New Zealanders of European descent) and Māori populations. Using qualitative interviews with three ethnic sub-groups in the Clutha District and Southland Region—the Filipino, Samoan, and Malay communities—we investigate their experiences of settling in small towns. While there is considerable variation in the experiences and aspirations of these ethnic minorities, for each community we demonstrate how local and regional contextual factors shape life aspirations, support infrastructures and settlement trajectories. Particularly drawing upon informal networks and social capital immigrants mediate the considerable challenges they face. Our study also demonstrates the limitations of current policy support and initiatives. Indeed, while local authorities clearly have a significant role in creating the conditions for enabling immigrant settlement in small centres in Southland-Clutha, now the role of government services and community-based support also needs to be considered.

## Introduction

As with many other OECD countries, over the last 40 years, rural and small-town New Zealand has experienced significant demographic and economic changes (Brabyn, 2017) through steady out-migration, falling birth rates and population ageing, neoliberal restructuring, including the ending of state support for agriculture and manufacturing, technological change and exposure to global markets since the 1980s/1990s. These changes have led to the emergence of the so-called “zombie” town discourse (Nel et al., 2019) and the associated “demographic disruption” of the predominantly Pākehā (New Zealanders of European descent) and indigenous Māori populations (Alam & Nel, 2023; Spoonley, 2020). While these changes might be seen as most likely reducing the population of small and regional towns, the situation is rather more complex in New Zealand’s South Island, as different regions have taken different development (and recovery) pathways. In some areas, the primary sector (e.g. dairy) has thrived relying on the immigrant workforce (Rawlinson et al., 2013). In other places, strong local leadership (e.g. Venture Southland, see Spoonley and Bedford (2008)) have helped retain services and infrastructure, which have helped facilitate amenity migration and tourism development, and partially replaced their historic “resource” dependency (e.g. on mining) (see Conradson & Pawson, 2009; Nel et al., 2019; Powe et al., 2022).

Indeed, changes in immigration policies since the 1990s have led to greater ethnic diversity in New Zealand’s immigrant population. While the majority of new immigrants have settled in the larger cities, where support is found from pre-existing ethnic networks (Maré et al., 2007), there are also immigrant flows to remote parts of the country, such as Southern New Zealand, as new immigrants respond to labour shortages in rural areas and small towns (Akbari & MacDonald, 2014; Alam & Nel, 2023). Some regional towns are gradually seeing a transition from near ethnic homogeneity to the slow emergence of what Woods (2018a) refers to as ‘cosmopolitanising’, where immigrants are now helping fill vacancies in the primary and secondary industry sectors, filling roles once played by the quintessential Kiwi^2^ farmer and small-town populations (Trafford & Tipples, 2012). Indeed, many immigrants now work in these areas because their previous experience qualifies them for visas as priority workers with skills that are in short supply. Patterns of international in-migration are not uniform across the whole of New Zealand, and it is often the more productive agricultural areas, such as the Clutha District and Southland Region, the focus of this study, where the more significant processes of cosmopolitanisation are occurring.

Despite recognition of these demographic and cultural shifts, studies examining how new immigrants settle in and develop a sense of belonging in regional New Zealand are scant. Fanselow (2019), Friesen (2015) and Spoonley and Bedford (2008) are a few notable exceptions; however, they largely focused on demographic changes and recruitment policies rather than on lived experiences. Arguably, this absence is partially attributable to the fact that mobility research has typically focused on large urban centres, which are conventionally framed as being “gateway” cities. Research in Australia and Canada on immigrant lived experiences is growing. There is evidence that many smaller centres continue to struggle in attracting and retaining immigrants (Stump, 2019) as immigrants often face significant challenges in them due to isolation, prejudice, difficult working conditions and harsh climate (Klocker et al., 2021). However, regional areas also create opportunities for immigrants with certain skills, such as farming to thrive and cultivate belongingness (Dun et al., 2018). Recent studies also focussed beyond ‘instrumentalist’ concerns such as employment, housing, and services to show that localised attributes, e.g. environments, climates, lifestyles, plants, and animals in small centres influence settlement experiences (Alam et al., 2020; El-Bialy & Mulay, 2015; Klocker et al., 2021). These works inspire us to examine particularly how place-specific factors shape immigrant settlement in small towns. Geographically we focus on the far south of New Zealand in the Southland Region and Clutha District, where ethnic immigrants are increasingly visible.

In what follows, we first engage with existing theories related to immigrant settlement in small towns which will later help us explain our findings from small town New Zealand. Following this, we briefly explain the trends of international migration to small-town in New Zealand to set the scene for our specific findings. We then provide contextual details about the study areas, the research methods used, the selection of participants from three ethnic sub-groups—the Filipino, Samoan and Malay communities—and the analysis of immigrant stories in line with the key questions: What are the processes and pathways individuals navigate to organise their lives in small towns? How do individuals make sense of their lives in small towns? How do the place-based dynamics of small towns shape the immigrant experience and what role do local institutions play in encouraging and supporting immigrants? In the final section, we explain how the findings from New Zealand speak about and contribute to existing scholarship, particularly the shared similarities with and differences to the experience of countries such as Australia and Canada. In the conclusion, we discuss a few unique place-based dynamics observed and future research considerations.

## Immigrant Settlement in Small Towns: a Theoretical Lens

International migration to rural areas and small towns has become an identifiable reality in many countries, leading to the emergence of what Woods (2007) refers to as the “global countryside” which is increasingly associated with the phenomenon of ‘rural cosmopolitanism’ (Woods, 2018b) and the emergence of the multicultural countryside (Wilding & Nunn, 2018). Despite the growing significance of this phenomenon, Bonifacio and Drolet assert that there has not been much scholarly attention given to “issues and challenges of immigration and diversity in communities beyond the metropolis” (2017, p. 3). The processes of rural change cannot be divorced from well-documented research into how, over recent decades, many small towns and rural areas in OECD countries have experienced rural out-migration, depopulation and economic contraction. The changes in turn have catalysed local development endeavours in small towns, aimed at local regeneration, a key element of which includes place-marketing and efforts to attract new-comers to such places (Woods, 2010; de Noronha Vaz and van Leeuwen, 2016; Nel et al., 2019). Within this context the potential which immigrants can play in small town regeneration and economic diversification is being increasingly recognised, leading to the emergence of defined strategies of small town development anchored on immigrant attraction (Hanley, 2017).

Causes for international migration, how the migration occurs, where and how immigrants settle, and whether they stay or move, are topics of growing academic interest. This is particularly in the Australian and Canadian literature, on which this and subsequent paragraphs in this section are based. Beyond the instrumentalist push-pull theories of migration (see Trafford & Tipples, 2012, pp. 19-20), small town studies pay growing attention to social capital and connections within immigrant communities and those which they have with the host society (Wulff and Dharmalingam, 2008; Weerasinghe et al., 2017). Equally important is social resilience when immigrants face challenges when settling, including friction with host communities and cultural clashes (Preston et al., 2022). Where immigrants have settled successfully and cultural acceptance occurs, there emerges meaningful “relationships (social capital) within and beyond ethnic communities” (Missingham et al., 2006, p. 144). Where immigrant settlement is successful, affective settlement and place-based belonging and place-making emerge, which can lead to the gradual emergence of new communities in place, characterised by diversity and integration (Boese & Phillips, 2017a). That said, particularly in some rural areas, racial friction and discrimination can also emerge and immigrants may struggle to settle, and retention is compromised (Leitner, 2012).

Settlement is often the outcome of “chain- or stepwise migration” when social and family connection and word-of-mouth lead to defined movement of family, clan and friend groups to a particular overseas destination, as has been noted in Australia (Missingham et al., 2006). In addition, immigration and settlement are shaped and supported by local institutions—official and voluntary—and can range from the support provided by cultural and ethnic groups, and faith-based groups to formal government structures (Alam & Nel, 2023; McAreavey & Argent, 2018). Local governments in particular can play key roles in local support and settlement processes (Boese and Phillips, 2017b).

Immigration patterns and settlement choice in certain countries are influenced by regionalisation policies—such as in Canada and Australia where rural and small-town depopulation has led to the enactment of policies which either through state support or differential immigration policies encourage migrants to settle in non-metropolitan areas (Krivokapic-Skoko and Collins, 2016; Hanley, 2017). Immigrant experiences however show that the attraction and slow pace of life in rural areas has to be balanced against the cost of living or remoteness and parochialism. That said, where settlement is successful, and new immigrants settle, it leads to ‘counter-urbanisation’ and helps restore rural vitality (Klocker et al., 2020), which is a core interest in this particular study. As work from Canada shows, successful settlement requires government support, educational and employment opportunities, successful integration, and access to a decent quality of life (Hanley, 2017).

## Recent Trends in Immigration to New Zealand

New Zealand’s gradual adoption of a more inclusive immigration policy from the 1990s (Ongley & Pearson, 1995) as a response to fluctuations in labour demand is not dissimilar from moves in Australia and Canada to address labour force challenges, particularly through easing restrictions on potential Asian immigrants. From the 1990s, an increasing focus was evident in government policy on skilled migrants from Asia, as well as a more recent renewed recruitment from the Pacific (Bedford et al., 2002). As such, it was in the 2000s, after the introduction of the skilled migrant category in 2003, that these population groups began to rise substantially. Indeed, between 1996 and 2018, the percentage of the national population who identified as being Asian rose from 4.4 to 14%, while the percentage of people who identified ethnic links with Pacific Island Countries (PICs) rose from 4.8 to 7.4% (Stats NZ, 2021).

As might be expected given the pace of these changes, literature has examined the impacts of shifts in immigration on New Zealand society at a range of scales from the national to the local. Despite a formal embrace of multiculturalism, some of this literature has questioned whether the country that has, until recently, identified itself as bicultural, is doing enough to support and welcome those who come from Asia and the Pacific (Annabell & Nairn, 2019; Spoonley, 2014). Other studies have focused on specific communities, with most of these centring upon the experience of immigrants coming to larger cities, or on specific industries that are reliant on immigrant labour, such as the dairy sector (Collins & Bayliss, 2020; Rawlinson et al., 2013).

Of the three ethnic groups chosen in this paper, Filipinos have received by far the greatest academic attention. Filipino migration to New Zealand began from a period in the 1980s and grew slowly throughout the 1990s. The trend accelerated after 2007 when the Philippines was designated a “comparable labour market”, so that training in that country was recognised by Immigration New Zealand, to the extent that from 2013/2014 onwards, Filipinos constituted the largest immigrant group within the essential skills visa category (Friesen, 2017, p. 280). Unsurprisingly, perhaps, given the increased significance of Filipinos within New Zealand’s economy and society, there have been an array of studies devoted to individual sectors, including nurses (Mowat and Jarrod, 2018), dairy workers (Bradford and Abel, 2017), construction workers (MacLennan, 2018), senior migrants (migrated after 60 years old) (Montayre et al., 2017) and high-skill immigrants (Siar, 2014).

Similar to many other countries, there is considerable evidence to suggest that new immigrants tend to settle in areas that contain dense immigrant networks (Maré et al., 2007). That said, Maré et al. (2007) point out that in New Zealand, well-established immigrants display tendencies to move to where economic opportunities are, as opposed to being strongly influenced to remain in parts of a large city where there are people of the same ethnicity. In addition, where there have been defined labour shortages, such as in the study area, employers have often recruited workers directly from particular overseas communities, such has been the case of Samoan workers in the meat works in Balclutha and dairy workers from the Philippines (PSN1/M/Balclutha). This, in recent years, has led to the emergence of small, skills-related ethnic clusters of new immigrants often associated with primary and secondary sector economic activity in smaller urban centres such as Invercargill, Mataura and Balclutha.

It is important to note that many immigrants have experienced a range of challenges following their move to New Zealand. These include the frequently reported experience of linguistic and cultural barriers, discrimination from employers and the reality for many that they have been unable to use their previously acquired qualifications, experiencing “downward occupational mobility” as a result (Feng & Page, 2000, p. 254). Grbic et al. (2010) also note the reality of labour and residential market segregation which people from the Pacific and Asia experience when moving to New Zealand. At the same time, many of those who came as temporary workers subsequently gained Permanent Residency, with midwifery and nursing, ICT and engineering, and farmers and farm managers being the most successful occupational categories for Filipinos (Friesen, 2017, pp. 282-3).

Compared to Australia and Canada, where federal governments have actively supported regionally focussed visa categories (Akbari & MacDonald, 2014), in New Zealand different temporary and seasonal visa schemes are related to specific skills required in the agriculture and other sectors that draw immigrants to regional places (Trafford & Tipples, 2012). Increased ethnic visibility in small towns without regionally focussed place-based policies led to challenges for immigrants settling in these less diverse parts of the country (Collins & Bayliss, 2020; Rawlinson et al., 2013). To tackle the challenges, local councils across Otago and Southland regions have become accredited with the “Welcoming Communities” initiative, and implemented settlement support within their long-term plans, aiming to “mobilise and involve local residents in welcoming activities” (Immigration New Zealand, 2017, p.3). There are 26 Welcoming Communities Councils in the country which are accredited and supported by Immigration New Zealand and the Ministry for Ethnic Communities. They are tasked to help new migrants settle in New Zealand and despite their small budgets and staffing levels, there is some evidence that Welcoming Communities Councils are playing a positive role in the settlement of new migrants (Fanselow, 2019). While more investment is needed in this area, as our research shows, it is often local migrant communities themselves who address shortfalls in settlement support.

## The Study Area: Southland Region and Clutha District

**Fig. 1:**
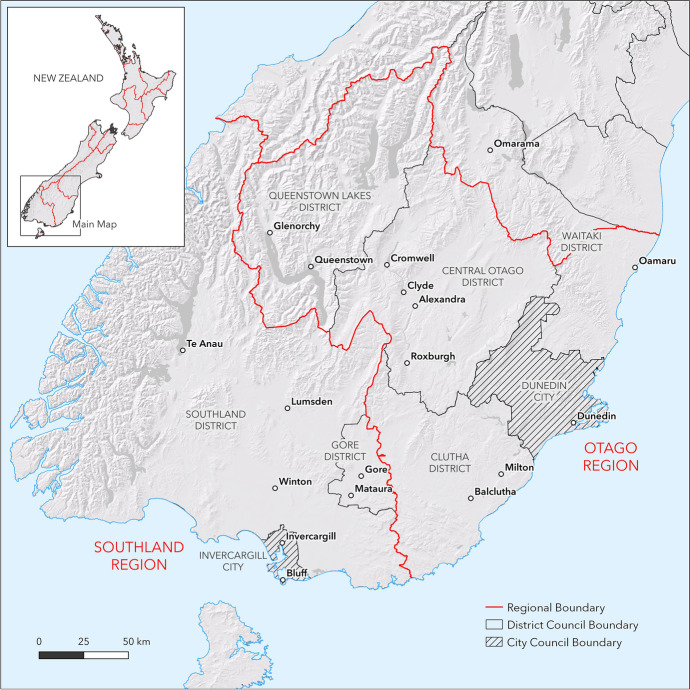
The study area: Southland Region and Clutha

This study focuses on the southernmost administrative regions in New Zealand—namely the Southland, Gore, Invercargill and Clutha district council areas. New Zealand’s local government system comprises two complementary sets of local authorities—regional and territorial authorities (i.e. the city and district councils). Administratively Gore, Southland and Invercargill are part of the “Southland Region”, while Clutha is part of the neighbouring ‘Otago Region’ (See Fig. 1 for administrative boundaries). These districts are considered together because of their similar demographic and economic histories, with all having an above average dependence on primary economic activity—namely farming and the processing of agricultural produce—and having experienced noticeable depopulation prior to 2006 but having grown significantly since then (Friesen, 2015).

Table 1 shows the degree to which the national population increased in the period up to and beyond 2006, while that of the Clutha District and Southland Region declined noticeably. In our study area, the falling population has caused significant local concerns regarding the directly associated labour shortages in the dairy and associated agro-industrial sectors (Grant, 2015; Tohill, 2017). Thereafter, two parallel processes—the steady growth in the primary sector as the main economic driver, and the low and often negative population—have attracted labour mobility to the region.

**Table 1:** Population change in the study area, 1996–2018^3^

	1996	2006	Change (%) 1996–2006	2018	Change (%) 2006–2018
New Zealand	3,681,546	4,027,947	9.4%	4,699,755	16,7%
Clutha District	18,006	16,839	-6.5%	17,667	4.9%
Southland Region	97,058	90,876	-6.4%	97,467	7.3%
Invercargill	53,208	50,325	-5.4%	54,204	7.7%

Source: Stats NZ Census

**Fig. 2:**
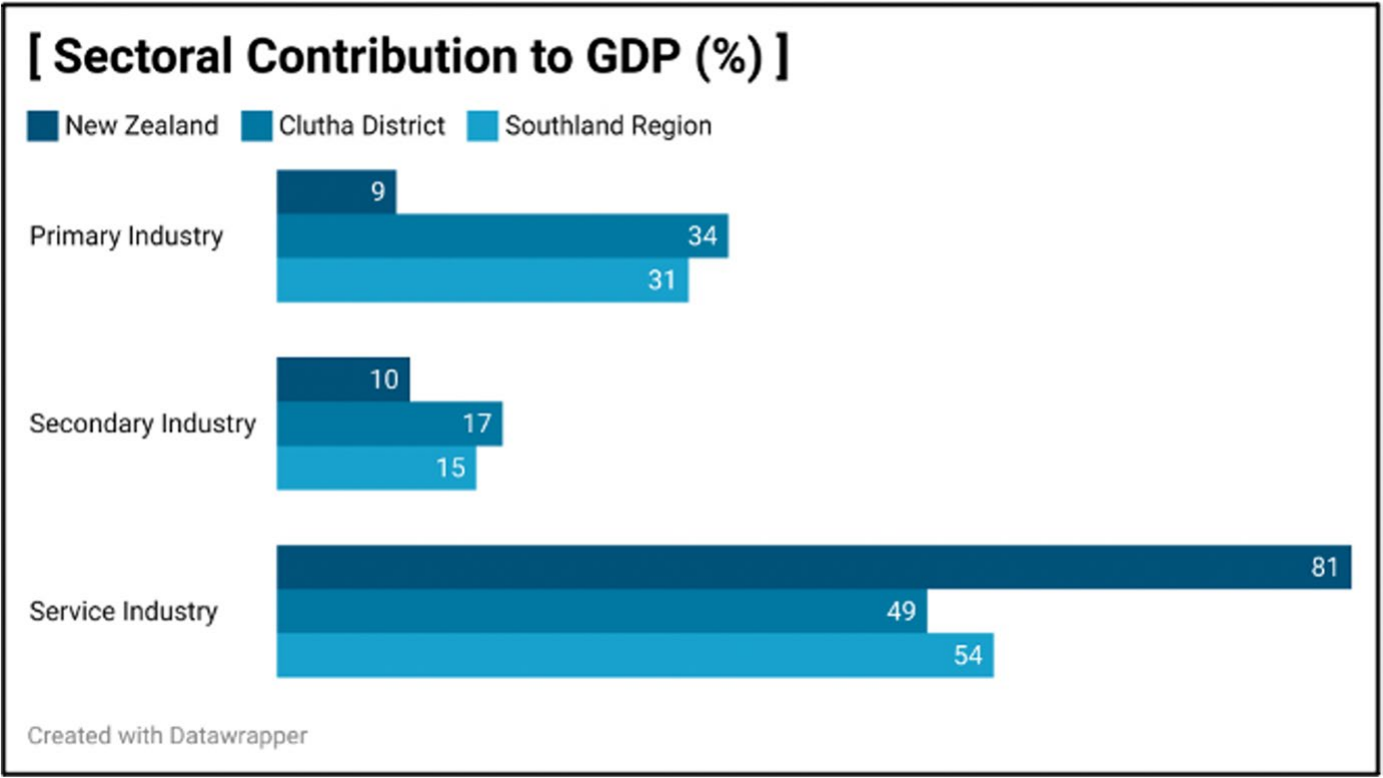
Sectoral Contribution to GDP (in %) in 2020

In both Clutha and the Southland, the main primary activities are dairy, beef and sheep farming and forestry, while the main secondary industries are processing of dairy, meat and timber (Fig. 2). Growing international demand for New Zealand’s dairy products from the 1980s drove significant conversion of farmland from sheep and beef production to intensive dairy farming. The already strong timber and animal processing industries were expanded, and numerous dairy processing facilities were constructed (Fonterra, 2014). From 1990 to 2013, the number of sheep fell from 8.9 million to 4.3 million and the number of dairy cattle rose from just over 37,000 to more than 615,000 (Stats NZ, 2014) creating increased labour demands which have been partially addressed through immigrant attraction strategies (Rawlinson et al., 2013).

Demographically, for most of the last 150 years, the population of the study area was extremely stable in terms of its ethnic composition, with over 85% of the population in 1996 being of European descent, while only 10–11% of the population were Māori, New Zealand’s indigenous population. This also meant that in numerical terms, there were virtually no other ethnic groups of significant size in the area (Cain et al., 2017). This began to change after 2000 as a result of labour demand, which led to not insignificant increases in the presence of ethnic minorities, but it also meant that for the first time, the study area was experiencing the need for the established, largely European population, to acknowledge and accommodate people of different ethnicities, with the latter needing to integrate into historically bi-cultural contexts (Friesen, 2015).

After 2006, the small, but significant in-migration of ethnic minority groups, often from overseas, helped to address labour shortages and reverse the population decline (Collins, 2021). Approved essential skills visas for migrants to the Southland Region rose from 873 in 2010 to 1, 518 in 2019. Cain et al. (2017) note that between 2001 and 2013 the percentage of the Southland population born overseas rose from 6.3 to 9.4%, with the largest increase of any single group being Filipinos. From 2012 the increased presence of overseas born workers in Southland was identified as an increasingly vital factor in the sustainability of local economic activity (Rawlinson et al., 2013). Initial signs from 2020/21 however, are that skills shortages in the agricultural sector are starting to grow again due to COVID-related restrictions on migration, even of skilled workers (Collins, 2021). From 2020 to 2022 border closures to all but returning residents and citizens temporarily halted the arrival of new migrants. This shows the weakness of the immigration regime that historically promoted high levels of temporary mobility without adequate long-term settlement and inclusion opportunities.

Table 2 indicates the changing ethnic profile of the population in percentage terms in New Zealand, in the Clutha District and the Southland region, and in selected primary and secondary industrial towns from 1996 to 2018. While at a national level, the Pacific and the Asian shares of the population increased significantly, even more striking increases can be observed in the study area. Broadly speaking the proportion of Pacific and Asian people increased often more than five-fold in 22 years in the study area, albeit from a very low initial base. For example, the percentage of the population in Balclutha identifying in the census as Asian increased from 0.1 to 6.4% of the town’s population, while the Pacific population grew from 0.7 to 3.3% of the population. These figures clearly indicate the degree to which the population of the study area is becoming more ethnically diverse; however, there are no distinctive immigrant residential clusters forming like “Little Manila” or “China Town” which are evident elsewhere.

**Table 2:** The composition of the resident population (in %) in terms of ethnicity

	New Zealand		Clutha District		Balclutha Town		Southland Region		Invercargill		Mataura		Winton	
	*1996*	*2018*	*1996*	*2018*	*1996*	*2018*	*1996*	*2018*	*1996*	*2018*	*1996*	*2018*	*1996*	*2018*
*European*	71.7	62.4	87.6	82.8	88	78	85	76.5	81.7	81	70.7	65.5	85.5	83
*Māori*	14.5	14.8	8	12.4	7.5	12.5	11	14.9	12.4	17.4	25.7	31.5	8.5	11
*Pacific*	4.8	7.4	0.5	1.9	0.7	3.3	1.2	2.6	1.9	3.8	0.3	2.8	0.2	1.8
*Asian*	4.4	14	0.7	3.6	0.1	6.4	0.7	5.5	0.8	5.7	0.4	2.2	0.1	2.7
*Other*	4.6	1.4	3.1	1.4	2.6	1.2	3	1.5	3.1	1.5	2.6	1.1	5.2	1.4

Source: Stats NZ Census^4^

The labour needs in our study areas provoked both policy and management response and the development of strategies to attract and retain potential new residents and workers to the area (Clutha District Council, 2015). Active settlement support and labour attraction activities have been pursued in Clutha and in Southland through the regional development agency, Venture Southland since the early 2000s (Clutha Development, 2018). Since 2017, the Welcoming Communities programme supported local councils and their communities in the region to create inclusive environments for newcomers (Fanselow, 2019). Nonetheless, the longer-term dynamics of successful immigrant retention in these areas are understudied and this paper goes some way towards filling this gap by qualitatively examining how the dynamics might vary between different ethnic groups.

## Study Participants and Methods

With a focus on first-generation immigrants of minority ethnic backgrounds,^5^ we were interested in the experiences of immigrant individuals and their family members (Table 3). All four authors were involved in the fieldwork between May and November 2021. The selected immigrant participants have lived in New Zealand for a significant duration, the earliest arriving in 2004 and the latest in 2018. This enabled participants to provide in-depth reflections on their post-arrival experiences and settlement focused on three stages of migration, namely: 1) pathways and aspirations that instigated migration to regional New Zealand, 2) challenges and opportunities of settling in regional locations, and 3) self-evaluation of migration decision and future plans.

**Table 3:** Immigrant sub-groups studied in three small towns

Immigrant sub-group	Interview locations	Population size	Primary religious affiliation	Industry sector involvement by participants	Data collection (details of numbers interviewed)
Samoan	Samoan Church, Balclutha	144 (Balclutha)^#^	Christian	Meat work and freezing industry	GI – 1(2) KI – 1
Filipino	Multiple locations, Invercargill City	543 (Invercargill city)^##^	Christian	Dairy, meat-work, agriculture, care work, education and other sectors	GI – 4(10) One-to-one interview – 4 KI – 4
Malay	Mataura Mosque	75 (Invercargill city, Clutha and Gore Districts combined)^##^	Muslim	Mostly in meat work, dairy, agriculture and hospitality sector, but there are also exceptions, e.g. medical doctor	GI – 3(11) KI – 2

Source: ^#^Stats NZ (2019), ^##^MfEC (2022)

Depending on the communal dynamics and availability of each ethnic sub-group, different combinations of interviews were adapted. Interviewees were identified through making contacts with ethnic / community groups and a purposive sampling technique was employed. In Mataura, we talked to eleven participants through two group interviews (GI) involving Malay male members and one with four females. One group interview was conducted in Balclutha that involved two male and female Samoans. For Filipino communities, we conducted four one-to-one interviews with three female participants and one male participant. Three group interviews involved both male and female household heads from the same family and one group interview involved four participants from two families.

All participants were of working age, ranging from their mid-to-late thirties to early-to-mid fifties. With two exceptions where one Malay participant entered New Zealand as a postgraduate student and a Filipino woman as a dependent partner to a New Zealand man, all Filipino and Malay participants entered as temporary labour migrants in the sectors detailed in Table 1 and some later gained permanent residency status. The pathway for Samoans is different because of the Samoan Quota Resident Visa which still required Samoans to have an offer of employment, either managed through recruitment agencies or through family relations.^6^ For Samoan and Malay communities, the key contact point (respectively, the church at Balclutha and the mosque at Mataura) limited the recruitment of participants in this study to those with dominant religious affiliations in these communities.

The COVID-19 pandemic at times made the fieldwork difficult and significantly limited the collection of narratives. To tackle this limitation, immigrant narratives were supplemented with existing academic and non-academic studies and with additional details collected through interviews (KI) with community leaders in each immigrant sub-group: the pastor in Samoan church at Balclutha, the *imam* and the president of the mosque at Mataura and three Filipino community group leaders in Invercargill city. A staff member from Southland’s regional development agency, Venture Southland, now Great South was also interviewed. All interviews were conducted in English as participants from all three ethnic groups presented conversational English language competence. Interviews were transcribed and interview data were coded to inductively identify key themes that emerged from participants’ descriptions of the three stages of migration.

It should also be noted that despite the small size of the selected settlements, because migrants come from many different nations, we were not able to fully capture the range of all migrant groups but rather focussed on the experience of the larger communities. In addition, the relatively small sample size means that our results reflect general trends and cannot be taken as representative of the experience of all migrants.

## Immigrants’ Experiences of Settling in New Zealand’s Small Towns

### Journeys Across the World, Journeys Across New Zealand

The interviews reinforced the notion that the study areas’ economic dependence on primary and secondary industry, and on agriculture and agricultural processing, has been a key migration pull factor for Filipino, Malay and Samoan immigrants. However, these are not unilineal journeys from an immigrant’s home country to a fixed place of settlement; instead, many participants fit the characterisation of stepwise migration, having had several different places of work and residence across the world and within New Zealand before arriving in Southland. For example, many of our participants from the Philippines first found work in the Gulf countries, and with experience in relevant sectors were then eligible to apply to move to New Zealand. Almost all our participants indicated that after entering New Zealand, they moved into small or mid-sized places close to large urban centres—such as Rotorua and Hamilton (near Auckland), Palmerston North (near Wellington) or Ashburton and Rakaia (near Christchurch)—because of the availability of work in agriculture. A few were initially drawn to big cities such as Auckland or Christchurch where there were large, established Filipino or Malay diasporas or extended Samoan family connections. Family connections providing the bridge to pursue new opportunities in New Zealand are particularly common to Pacific Country citizens.

Initially, then, the patterns of immigrant journeys accord with literature suggesting that ethnic connections and social capital significantly influence the settlement decisions of immigrants and assist their employment integration, with previous literature finding that these connections are particularly significant for the settlement decisions of those moving from non-English speaking backgrounds (Cassim et al., 2020). Thereafter, many moved to Southland-Clutha in search of a better work-life balance, affordable housing, and strong employment prospects in areas of their skill sets, all contributing to the potential for finding a “good life” in a small town, as the next section describes. According to a Filipino participant now based in Invercargill,

Small towns are good places to start off, to raise children and set the footing, you know; they can find a proper home early into their childhood. Besides, I have plenty of time which I couldn’t afford to give them when I was stuck in Auckland’s traffic. [PF3/M/Invercargill]

### Good Life Aspiration and Settling in Small Towns

Our participants, with their experiences in several countries and regions within New Zealand before relocating to Southland-Clutha, envisage New Zealand as a desirable destination because of the opportunities for gaining permanent residency and, ultimately, citizenship. Participants suggested that similar long-term opportunities were limited in places like Korea, Singapore, or Saudi Arabia where they had lived and worked before. As reinforced by participants, many international labour migration destinations do not enable workers to bring partners and children; and the fact that New Zealand immigration regulations allow for family visas including free primary and secondary schooling for children is a major attraction.

As such, New Zealand, and particularly its small towns, seemed to offer several practical and subtle benefits of locating to small centres, relating to long-term good/better life aspirations, lacking elsewhere. Obviously, economic opportunities in sectors appropriate to their knowledge, qualifications and skill sets were the key driver. Extended job contracts (tenure security) and stability in the employment sector contributed to their residency visa acquirement in the long run.^7^

Almost all participants said they moved to regional locations because of work, and later they found these areas catered to their good life aspirations by providing some degree of affordances in their everyday life in areas that large urban centres could not offer. One key attractor is the relative housing affordability that was “unimaginable” in places like Auckland. Cheaper housing costs enabled savings in the early stage of migration, which enabled people to purchase a house relatively earlier than their peers in Auckland or Wellington. Especially for families with children, higher housing prices in larger centres meant an inevitable compromise in the amount of housing space, and some saw this as counterproductive to good family life as children would suffer from a lack of adequate domestic space (e.g. of required number of bedrooms, backyard etc.). Multiple members from the Malay and Filipino communities expressed the view that small towns were conducive to their search for the ‘type’ and ‘quality’ of life that they thought was impossible in metro Manila or Kuala Lumpur. PM1, who had migrated in 2004 and stayed in the small town of Roxburgh since 2005, said,

I was dreaming of the green pasture… I never wanted to be in a busy place because you know, if I looked for that, what’s the point of leaving Kuala Lumpur? I had business over there inside the mall, I came here to do skiing, hiking, surfing, tramping – all those things Kiwi people do. In the end, I wanted peace in life, the very first time I stepped in Roxburgh, I knew, this was the place. And I’m staying there since then. [PM1/M/Mataura]

### Smallness of Small Towns Boosting Settlement Attraction

Participants also spoke of the importance of the smallness of their adopted settlements in contributing to their social connections and ease of mobility. One obvious advantage of staying in small towns was that it was relatively easy for some to strategically position themselves in the workplace and from there plan for the long term. Many participants suggested that they felt “lost” in bigger centres—“I lived in Auckland for ten years; there, people do not say ‘hello’ anymore” [PM3/M/Mataura]. There was a lack of the sense of “recognition” of their “good work” both at their workplaces and within their own immigrant community as those places were too ‘busy’ to get “noticed” which previous studies (Spoonley et al., 2005) suggest would otherwise reinforce the sense of belonging to destinations. Multiple Filipino participants suggested that the small and intimate nature of these settlements helped them to establish trusted long-term relationships with their employers.

Opportunities for re-skilling, particularly for dependent spouses/partners, were perceived as easier because of the relative accessibility to education (such as the Southland Institute of Technology, a technical college in the study area). This was further reinforced by a high level of motivation that many expressed, e.g. “I am here to find a good life, I must assimilate, I must work towards that” (PF4/F/Invercargill). This drive to work and try hard to integrate and not challenge any abuse they may face in the workplace is not uncommon among first-generation immigrants. In particular, this is a strong discourse among the Filipino diaspora. As one participant, PF1 (F/Invercargill), reflected, “I told my children, if you do not like something (work), love it”. Small towns worked well for these immigrants who were desperate enough to prove their worth to employers and build a reputation in their communal circles. Having the relative luxury of time in small towns to explore alternative career pathways also coincided with such perseverance. One participant recollects her family’s hard work in the last sixteen years, saying,

Compared to where (Ashburton and Timaru) we started first, these places worked well for us… my husband started as a share milker, then he could move up to become a contract milker and then at one point, he became farm manager of the year. We are hard workers, you know… I started as a farm labourer, then a store worker, to a young nurse to now a care home manager. We now have a place in this society. [PF4/F/Invercargill]

### Small Towns Boosting Self-organising Settlement Support

Compared to Auckland or other bigger cities, where there are more formalised support systems available within established immigrant clustering, small-town ethnic communities have to self-organise small support networks. For example, the Malay community has established their own support system built around the mosque at Mataura, established in 2017. In the absence of visible residential clusters, the families—who were spread across several regional centres, including Mataura, Gore, Roxburgh, Invercargill, and Queenstown—congregated usually once a month for afternoon and evening prayers. Between the two prayers, they shared ethnic cuisine, and the *Imam* led a session to give the young members greater clarity around religious and cultural boundaries (such as *halal* food and practices). One participant explained the importance of this regular gathering,

This is not just for prayer. If you want to pray, you have a mosque in Invercargill. Why bother driving for an hour to Mataura? It’s like a cultural evening: you share your own food, the children get to meet elderly members and learn etiquette from the *Imam* – like what you can’t eat, what you wear, but most importantly, how you also become a good Kiwi. We deliberately use English in the session, which the children understand better. [PM3/M/Mataura]

Two of the authors attended a similar gathering by the same community in Invercargill during July 2021, celebrating the Eid-ul-Adha festival. These gatherings seemed key in educating the younger generations how to navigate multiple identities in Aotearoa as they settle in, to help ensure their Malay and Islamic identities are not in conflict with their Kiwi identities. As we heard the *Imam* reinforce during his preaching, “fulfill the obligations of our religion to the point that they do not go against the law of the land.” [PM4/M/Mataura]

Both Filipino and Samoan community networks devised strategies to meet their cultural needs. These often centered around specific churches and religious leaders in Invercargill and Balclutha. A number of subgroups within the Filipino communities organised activities like dance classes, cooking lessons, badminton sessions, etc. that were supported around members’ affiliation with a particular church. These church groups also provide more pastoral care to the members in educating them how to settle in smoothly, as explained by the religious leader in Balclutha,

I know every family, and many of them need education – how to maintain the kitchen, the garden, you have to mow your lawn twice a week in this part of New Zealand. We organise sports. Rugby plays a big part for a Samoan to find comfort. We advise on tenancy dispute, police, visa, KiwiSaver [government supported superannuation scheme], how to budget, save for buying house… educating them about small town norms is important, you can’t drink drive and yell on the street here, you know. [PSN1/M/Balclutha]

### Yet, Settling Into Small and Regional Towns Has Unique Limitations

Despite the many opportunities participants shared about being in small towns, they also recognise limitations. It is recognised that participants need to make careful choices of employment pathways regarding what these places can realistically offer for long-term settlement. Participants were aware that young people, the second-generation members, may not aspire to the type of work their parents do, nor might these regional places accommodate their broader life aspirations. The Filipino woman [PF0/F/Balclutha], who migrated in 2013/2014, mentioned, “I am now planning to study at the university again. After that I may leave this place…” that would be able to cater to her newly acquired skills.

Literature has consistently demonstrated vulnerabilities and exploitation, most notably among those on temporary work visas and particularly those employed on dairy farms (Trafford & Tipples, 2012). As evident in interviews, workers on farms are sometimes subject to verbal abuse. They struggle to adapt to the English dialect used in this part of rural New Zealand and can suffer from social isolation, particularly if they are living a significant distance away from any towns and long working hours mean that it is difficult for them to leave the property. Within the largely white population that dominates regional areas, dairy workers are subject to racism and cultural stereotyping (see Collins & Bayliss, 2020). A notable source of employment for immigrant partners/spouses is in aged care facilities, and prejudice is also common there, as one participant explained,

We accept that in the care homes, the older generations, those who are more than eighty years old, are not comfortable with us. But you must also understand that at their time, they did not see many immigrants around them, so it is hard for them to accept. But over the years, these workplaces learned from us that they need to speak slowly. They need to embrace differences. Immigrants are the backbone of the country. [PF4/F/Invercargill]

Nonetheless, the potential of participants to imagine a permanent life in Southland-Clutha was undoubtedly complicated by their visa status. Indeed, while many became permanent residents, some of our participants struggled to gain residency and instead continue temporary work visas for an extended period. The longest waiting time among our participants was PF7 (M/Invercargill), who gained permanent residency, 16 years after his first arrival to New Zealand. This was a source of considerable frustration for many participants. The regulatory limbo that many immigrants found themselves in is attributable to New Zealand’s immigration policies, rather than a reluctance on the part of employers to sponsor migrants for permanent residency.

## Institutional Responses to Immigrant Settlements in Small Towns

Recognising the key role immigrants play in addressing employment gaps in the region, employers and regional and local authorities have pursued settlement support programmes to attract new immigrants and help them to settle. For example, Southland’s regional development agency, Great South (formerly known as Venture Southland), developed support initiatives and coordinated the Southland’s Welcoming Community Programme (Franselow, 2019). That said, in general, funds were only available for one person in that support role, with that individual being in contact with 850 migrants (Devlin, 2013a, 2013b). Local governments have generally played only a modest role in “settlement support”. Limited funds and personnel have meant that while migrants may receive advice and be invited to participate in occasional community events, they are not receiving the ideal “wrap-around” support. In our interviews, most participants did not mention institutional forms of settlement support as impacting their settlement experience and this is probably reflective of limited institutional resources. Rather it is the “non-formal” and small-scale community initiatives which provide more meaningful support to these ethnic immigrants.

From the perspective of our participants, the lack of explicit assistance from local government has been partially filled by local not-for-profit and community organisations, such as “multi-ethnic councils” and faith-based organisations, which have proven themselves critical in providing points of contact, support and social connections for Asian and Pasifika immigrants to New Zealand. Indeed, each of the three communities demonstrates how these networks function across different temporal and spatial scales to assist settlement.

The Malay community in the region are oriented around the mosque at Mataura, even though the families are spread across different medium to small centres. They have developed their own informal self-help support system, which assisted the process of settling in, and provided a sense of belonging to the Malay community. This is because of the multiple layers of connection that the mosque can provide in terms of language, culture, religion and identity, while also providing a mechanism to enable new immigrants to better understand and transition into the host society.

For the Samoan immigrants to Balclutha, it was apparent that the first batch of immigrants received settlement support from the local labour migration agent which had recruited them in response to local labour shortages. Over time, however, such support waned, and later arrivals floundered in the absence of support to cope with unfamiliar challenges such as finding accommodation and budget planning. While the local council was sympathetic to their plight, it lacked resources to provide significant support (Tohill, 2018). The arrival of a Samoan pastor and his wife, however, proved critical to recent Samoan immigrants. In addition to setting up a church, the couple helped move Samoan families from the squalor in which many were living – described as whole families living in single rooms—to new accommodation, often with the assistance of faith-based organisations in the town (PSN1/M/Balclutha). Additional support on budget planning, advice on living in New Zealand, the provision of social connections, and sporting and community events have, in particular, helped transition the Samoan community into a position of greater social acceptance in the town (PSN1/M/Balclutha; PSN2/F/Balclutha).

A notable feature of how Filipino immigrants settle in Southland is the extensive formal and informal networks they are able to draw upon, both on arrival and in subsequent years. Indeed, as has been found in other parts of New Zealand, the Filipino community have developed strong institutional support through a range of Filipino immigrant and multicultural organisations. The most notable of these in Invercargill is the Southland Filipino Society Inc. (SFSI), where long-term residents support the Filipino community in a range of ways, including providing information and mediation on employment issues, advocacy on immigration policy, and organising cultural events such as Philippine Independence Day celebrations. As most Filipinos are Christian, many also attend pre-existing churches in the region, and this (alongside their workplaces) is an important point of linkage with the broader community.

## Discussion and Conclusion

The study revealed the degree to which one region in New Zealand has experienced, for the first time, significant levels of ethnic diversity because of new immigrants filling gaps left by historic population contraction and expanded economic activity. The new arrivals display typical characteristics of immigrants, i.e. stepwise migration, and connecting with familiar ethnic networks. That said, the scale of the small centres has played a key role for immigrants in satisfying a range of their material and immaterial needs. This was particularly important for them to affirm that these places could meet their long-term settlement aspirations, therefore, they could take action accordingly by reskilling themselves, and enacting informal support groups and so forth. That said, this data does contain considerable silences and absences with regard to the multiple spatialities and temporalities of immigrant journeys in New Zealand, and it does not account for the clear occupational shifts that occur during the course of settling in small towns. While immigrants generally perceive the move to the small towns in a positive light and a move which improves their life prospects, there are some unique place-based dynamics which were observed.

Firstly, despite the initial good life aspirations and skill based economic opportunities that small towns were seen to meet, in the long run, small towns seem to fall short in meeting immigrants’ diverse occupational choices, particularly for the second-generation immigrants, as well as the foreign-born subjects themselves if they have a changed occupational interest after gaining permanent residency. That said, from a place-based perspective, most immigrants satisfy their needs by relying on the opportunities offered by the region that include multiple small towns and farming districts as an assemblage of diverse options.

Secondly, despite the increasing cosmopolitanisation of small towns and immigrants’ significant contributions to the regional economy, immigrants still need to navigate through forms of discrimination and racial stereotyping that are entrenched within these towns’ culturally homogeneous past and the imagination about these places belonging to the quintessential ‘Kiwi’ populations. These negative experiences, in many cases, stay clandestine, normalised, and are exacerbated due to migrants’ prolonged temporary visa status tied to their employment. Thirdly, ethnic immigrant settlement in small towns requires tailored support; however, the in situ institutional support, although increasing in recent years, has yet to cater to the culturally diverse immigrant aspirations. Therefore, immigrant sub-groups go on self-organising their support systems to address gaps in the institutional service provision. The smallness of small towns helps find like-minded peers with similar needs, and they build necessary social capital and communication from thereon.

Theoretically, our findings reveal important nuances beyond the instrumentalist reasoning behind immigrants' entry and settlement processes in small towns. We show how place-based factors such as environments, lifestyle and, most importantly, social connections shape immigrant entry, settlement support and retention and how the scale of small towns shapes these processes. Social connections were found as an important element in immigrant well-being, as has been observed elsewhere in Australia and Canada (e.g. Wulff and Dharmalingam, 2008; Weerasinghe et al., 2017). As found in Europe, small towns play an important role in defining immigrants’ perception of the destination country, immigrant identity and the idea of citizenship (e.g. Triandafyllidou, 2008). We also find that small towns influence the ways ethnic sub-groups organise themselves in New Zealand by carefully negotiating their religious, ethnic, and transnational identities. In our case, we see immigrants' non-formal, self-organising and sometimes faith-based social connections and associated cultural practices (e.g. cooking, sports, etc.) reinforcing both immigrant and Kiwi identities. We also observed that small towns are places of racial friction and discrimination that affected immigrant settlement experiences [also observed elsewhere, see Leitner, 2012]; however, the situation is improving as increasing ethnic diversity is becoming visible, and is therefore, being normalised as our participants suggest. Future studies should examine how ethnic immigrants negotiate their identities and place in the course of their settling in the bi-cultural New Zealand society which is different to that in Australia and Canada.

To conclude, this was the first study in the context of small-town New Zealand that looked into immigrant first-hand experiences by going beyond exploring the macro-economic push-pull factors of migration often with the conceptual foci on the labour sector and broader demographic questions. The findings demonstrate the different local and regional contextual factors contributing to the settlement of immigrants with distinct ethnic backgrounds and how these places fulfill their personal, familial, and communal needs. The findings can be helpful for immigrant inclusive policy formulation, regionalisation of immigration schemes, and other time-based labour migration schemes. Nevertheless, what was perhaps surprising to establish was that despite genuine labour shortages and modest efforts by local institutions to provide settlement support and immigrant attraction, we see individual choices, ethnic connections, and visa status as far more influential on immigrant settlement. As a result, while place-based needs exist in terms of labour, and these seem to be addressed over time, this is seemingly a process in which local institutions play a minimal role, a situation that merits attention going forward. The paper has highlighted that small towns with little history of ethnic immigration in the recent past are seeing the emergence of distinct ethnic sub-groups and as such is an important contribution to understanding how New Zealand regional cosmopolitanism is taking shape.

## Notes

1. There was a drop-off in the number of foreign citizens entering New Zealand between March 2020 and Dec 2021 (MBIE, 2022a) due to COVID-19-related border closure.
2. ‘Kiwi’ is colloquial for New Zealanders but is often taken to mean European New Zealanders.
3. Because of their relatively small size and integrated nature, data for the Southland, Gore and Invercargill Districts has been combined in Table 1, with urban Invercargill presented separately as the largest centre in the study.
4. As the census indicates, it is not uncommon for respondents in census counts to identify with more than one ethnic group, which can lead to overcounting. As such data is used to indicate trends (in percentages) rather than reflecting absolute numbers.
5. In terms of the major ethnicities, the New Zealand Census classifies people as ‘European, “Māori”, “Pacific”, “Asian” and “Other”. In this regard “Pacific” are people who were born in or identify with the Pacific Islands; in the Southland context, this refers primarily to people from Samoa, Fiji, the Cook Islands, and Vanuatu. The category of “Asian”, while collectively referring to anyone born in or of Asian descent, in the study area refers primarily to people from China, the Philippines, and Malaysia (Friesen, 2015).
6. Samoan Quota Resident Visa allows 1,100 Samoans to settle annually in New Zealand provided that, 1) a principal applicant (aged 18-45) must have a job offer that pays enough to support the individual including the accompanying partner and dependent children aged 24 and under, and 2) conditions and expectations, such as language proficiency outlined in the Pacific Access Category Visa are met (MBIE, 2022b).
7. While having “extended job contracts” is an important determinant for a Permanent Resident visa, some participants had lengthy waiting periods to gain residency, which has implications for their wellbeing and may encourage further migration to Canada and Australia (Flaws, 2021).

## References

[CR1] Akbari AH, MacDonald M (2014). Immigration policy in Australia, Canada, New Zealand, and the United States: An overview of recent trends. International Migration Review.

[CR2] Alam A, McGregor A, Houston D (2020). Neither sensibly homed nor homeless: re-imagining migrant homes through more-than-human relations. Social & Cultural Geography.

[CR3] Alam, A., & Nel, E. (2023). Migration, emerging multiculturalism and planning in rural and small town Aotearoa New Zealand. *Australian Planner*, 1–11. 10.1080/07293682.2023.2169724

[CR4] Annabell T, Nairn A (2019). Flagging a ‘new’ New Zealand: the discursive construction of national identity in the Flag Consideration Project. Critical Discourse Studies.

[CR5] Bedford R, Ho E, Lidgard J (2002). *International migration in New Zealand: context, components and policy issues*.

[CR6] Boese M, Phillips M (2017). Half of myself belongs to this town’: conditional belongings of temporary migrants in regional Australia. Migration, Mobility, & Displacement.

[CR7] Boese M, Phillips M (2017). The role of local government in migrant and refugee settlement in regional and rural Australia. Australian Journal of Social Issues.

[CR8] Bonifacio GT, Drolet JL (2017). *Canadian perspectives on immigration in small cities*.

[CR9] Brabyn L (2017). Declining Towns and Rapidly Growing Cities in New Zealand. Policy Quarterly.

[CR10] Bradford S, Abel S (2017). *In Search of Decent Work: Voices from Workers in the New Zealand Dairy Farm Industry*.

[CR11] Cain T, Peace R, Spoonley P, Pereda P, Vague P, Howard C (2017). *Population Change and its implications for Southland*.

[CR12] Cassim S, Hodgetts D, Stolte O (2020). Exploring distancing among Sri Lankan migrants in New Zealand. International Journal of Intercultural Relations.

[CR13] Clutha District Council (2015). *Living and Working in the Clutha District*.

[CR14] Collins FL, Bayliss T (2020). The good migrant: Everyday nationalism and temporary migration management on New Zealand dairy farms. Political Geography.

[CR15] Collins, F. (2021). Temporary migration in Invercargill and Queenstown amidst the COVID-19 global pandemic, CaDDANZ, Capturing the Diversity Divident of Aotearoa/New Zealand, Brief No. 12, University of Waikato, Hamilton.

[CR16] Conradson D, Pawson E (2009). New cultural economies of marginality: revisiting the West Coast, South Island, New Zealand. Journal of Rural Studies.

[CR17] de Noronha Vaz T, van Leeuwen E (2016). *Towns in a rural world*.

[CR18] Devlin, C., (2013a). Plan to stress need for migrant support http://www.stuff.co.nz/southland-times/news/9507205/Plan-to-stress-need-for-migrant-support

[CR19] Devlin, C., (2013b). Support service changes slap in the face http://www.stuff.co.nz/southland-times/news/9445651/Support-service-changes-slap-in-face

[CR20] Development C (2018). *Annual Management Report*.

[CR21] Dun O, Klocker N, Head L (2018). Recognising knowledge transfers in ‘unskilled’and ‘low-skilled’ international migration: insights from Pacific Island seasonal workers in rural Australia. Asia Pacific Viewpoint.

[CR22] El-Bialy R, Mulay S (2015). Two sides of the same coin: Factors that support and challenge the wellbeing of refugees settled in a small urban centre. Health & Place.

[CR23] Fanselow, M. (2019). Evaluation of the Welcoming Communities Pilot Programme. *Final Evaluation Report. MBIE*. https://www.immigration.govt.nz/about-us/what-we-do/welcoming-communities/resources-welcoming-communities/welcoming-communities-evaluation-final-report-2019-1.pdf Accessed 24 Jan 2022.

[CR24] Feng K, Page SJ (2000). An exploratory study of the tourism, migration–immigration nexus: travel experiences of Chinese residents in New Zealand. Current Issues in tourism.

[CR25] Flaws, B. (2021). Farmers celebrate migrant workers’ ‘well deserved’ fast-track residency. https://www.stuff.co.nz/business/farming/126537934/farmers-celebrate-migrant-workers-well-deserved-fasttrack-residency Accessed 24 February 2022

[CR26] Fonterra (2014) *Fonterra revises forecast farmgate milk price for 2014/15 season and announces estimated dividend.* http://www.fonterra.com/global/en/hub+sites/news+and+media/media+releases/fonterra+revises+forecast+farmgate+milk+price+for+201415+season+and+announces+estimated+dividend/fonterra+revises+forecast+farmgate+milk+price+for+201415+season+and+announces+estimated+dividendG Accessed 24 Feb 2022

[CR27] Friesen, W. (2015). Beyond the metropoles: The Asian presence in small city New Zealand. Retrieved from https://www.asianz.org.nz/assets/Uploads/Beyond-the-Metropoles-The-Asian-presence-in-small-city-New-Zealand.pdf

[CR28] Friesen W (2017). Migration management and mobility pathways for Filipino migrants to New Zealand. Asia Pacific Viewpoint.

[CR29] Grant, D. 2015) Southland region – Southland people, https://teara.govt.nz/en/southland-region/page-13 Accessed 14 November 2021.

[CR30] Grbic D, Ishizawa H, Crothers C (2010). Ethnic residential segregation in New Zealand, 1991–2006. Social science research.

[CR31] Hanley J, Bonifacio GT, Drolet JL (2017). The ‘Regionaization’ of Immigration in Quebec. *Canadian perspectives on immigration in small cities*.

[CR32] Klocker, N., Dun, O., Head, L., & Gopal, A. (2020). Exploring migrants’ knowledge and skill in seasonal farm work: more than labouring bodies. *Agriculture and Human Values, 37*, 463–478. 10.1007/s10460-019-10001-y

[CR33] Klocker N, Hodge P, Dun O, Crosbie E, Dufty-Jones R, McMichael C, Radford D (2021). Spaces of well-being and regional settlement: International migrants and the rural idyll. Population, Space and Place.

[CR34] Krivokapic-Skoko B, Collins J (2016). Looking for rural idyll ‘down under’: international immigrants in rural Australia. International Migration.

[CR35] Immigration New Zealand (2017). Welcoming Communities – Standard for New Zealand. Accessed from https://www.immigration.govt.nz/about-us/what-we-do/welcoming-communities/resources-welcoming-communities/welcoming-communities-standard.pdf on 16 Nov 22

[CR36] Leitner H (2012). Spaces of encounters: Immigration, race, class, and the politics of belonging in small-town America. Annals of the Association of American Geographers.

[CR37] MacLennan, C. (2018) Migrant Filipino Workers in the Construction Industry. A report for E Tū, funded by the New Zealand Industrial Relations Foundation (Inc).

[CR38] Maré DC, Morten M, Stillman S (2007). Settlement patterns and the geographic mobility of recent migrants to New Zealand. New Zealand Economic Papers.

[CR39] MBIE (Ministry of Business, Innovation and Employment) (2022a). https://mbienz.shinyapps.io/migration_data_explorer/# Accessed December 11 2022.

[CR40] MBIE (Ministry of Business, Innovation and Employment) (2022b). https://www.immigration.govt.nz/new-zealand-visas/apply-for-a-visa/about-visa/samoan-quota-scheme-resident-visa Accessed 25 January 2022.

[CR41] MfEC Ministry for Ethnic Communities (2022). Ethnic Communities in New Zealand. https://www.ethniccommunities.govt.nz/resources-2/ethnic-communities-in-new-zealand/

[CR42] McAreavey R, Argent N (2018). Migrant integration in rural New Immigration Destinations: An institutional and triangular perspective. Journal of Rural Studies.

[CR43] Missingham B, Dibden J, Cocklin C (2006). A multicultural countryside? Ethnic minorities in rural Australia. Rural Society.

[CR44] Montayre J, Neville S, Holroyd E (2017). Moving backwards, moving forward: The experiences of older Filipino migrants adjusting to life in New Zealand. International Journal of Qualitative Studies on Health and Well-being.

[CR45] Mowat R, Jarrod MH (2018). Sacrifices, benefits and surprises of internationally qualified nurses migrating to New Zealand from India and the Philippines. Nursing Praxis in New Zealand.

[CR46] Nel E, Connelly S, Stevenson T (2019). New Zealand's small town transition: The experience of demographic and economic change and place based responses. New Zealand Geographer.

[CR47] Ongley P, Pearson D (1995). Post-1945 International Migration: New Zealand, Australia and Canada Compared. International Migration Review.

[CR48] Powe NA, Connelly S, Nel E (2022). Planning for small town reorientation: Key policy choices within external support. Journal of Rural Studies.

[CR49] Preston V, Shields J, Akbar M (2022). Migration and resilience in urban Canada: Why social resilience, Why now?. Journal of International Migration and Integration.

[CR50] Rawlinson P, Tipples R, Greenhaigh I, Trafford S (2013). *Migrant workers and growth of the dairy industry in Southland, New Zealand*, Centre of Excellence in Farm Business Management – One Farm Management.

[CR51] Siar SV (2014). Highly skilled migrants’ strong ties with their home country: Evidence from Filipinos in New Zealand and Australia. Journal of International Migration and Integration.

[CR52] Spoonley P (2014). New diversity, old anxieties in New Zealand: the complex identity politics and engagement of a settler society. Ethnic and Racial Studies.

[CR53] Spoonley P (2020). *The New New Zealand: Facing demographic disruption*.

[CR54] Spoonley P, Bedford R (2008). Responding to regional labour demand: International migration and labour markets in New Zealand’s regions. Journal of International Migration and Integration.

[CR55] Spoonley P, Peace R, Butcher A, O’Neill D (2005). Social cohesion: A policy and indicator framework for assessing immigrant and host outcomes. Social Policy Journal of New Zealand.

[CR56] Stats NZ (2014). *Group: Agriculture – AGR001AA.* Available at: http://www.stats.govt.nz/infoshare/SelectVariables.aspx?pxID=6daab0fb-21c8-4cda-9587-799e52a2ecf0. Accessed 17 August 2015

[CR57] Stats NZ. (2019). 2018 Census place summaries. Retrieved from http://www.stats.govt.nz/tools/2018-census-place-summaries/

[CR58] Stats NZ (2021). 1996 and 2018 Census: https://www.stats.govt.nz/topics/census. accessed 7 November 2021

[CR59] Stump, T. (2019). The Right Fit: Attracting and retaining newcomers in regional towns. City to Country Project 10.13140/RG.2.2.34596.01927

[CR60] Tohill, M. (2018). Silver Fern Farms Finegand still looking at worker accommodation options https://www.stuff.co.nz/southland-times/news/108032258/silver-fern-farms-finegand-still-looking-at-worker-accommodation-options

[CR61] Tohill, M-J. (2017). Clutha District failing to attract permanent residents to fill hundreds of jobs, https://www.stuff.co.nz/southland-times/news/95863549/clutha-district-failing-to-attract-permanent-residents-to-fill-hundreds-of-jobs Accessed 14 November 2021

[CR62] Trafford S, Tipples R (2012). A foreign solution: the employment of short term migrant dairy workers on New Zealand dairy farms.

[CR63] Triandafyllidou A (2008). Popular perceptions of Europe and the Nation: the case of Italy. Nations and Nationalism.

[CR64] Weerasinghe S, Dobrowolsky A, Gallant N, Tastsoglou E, Akbari AH, Barber PG, Quaicoe L (2017). Why networks matter and how they work? The role of social networks in attracting and retaining immigrants in small cities. *Canadian perspectives on immigration in small cities*.

[CR65] Wilding R, Nunn C (2018). Non-metropolitan productions of multiculturalism: refugee settlement in rural Australia. Ethnic and Racial Studies.

[CR66] Woods M (2007). Engaging the global countryside: globalization, hybridity and the reconstitution of rural place. Progress in Human geography.

[CR67] Woods M (2010). *Rural*.

[CR68] Woods M (2018). Rural cosmopolitanism at the frontier? Chinese farmers and community relations in northern Queensland, c. 1890–1920. Australian Geographer.

[CR69] Woods M (2018). Precarious rural cosmopolitanism: Negotiating globalization, migration and diversity in Irish small towns. Journal of Rural Studies.

[CR70] Wulff M, Dharmalingam A (2008). Retaining skilled migrants in regional Australia: The role of social connectedness. Journal of International Migration and Integration.

